# A novel type of phytosulfokine, PSK-ε, positively regulates root elongation and formation of lateral roots and root nodules in *Medicago truncatula*

**DOI:** 10.1080/15592324.2022.2134672

**Published:** 2022-11-10

**Authors:** Qi Di, Yi Li, Danping Zhang, Wei Wu, Lin Zhang, Xing Zhao, Li Luo, Liangliang Yu

**Affiliations:** a Shanghai Key Laboratory of Bio-energy Crops, Plant Science Center, School of Life Sciences, Shanghai University, Shanghai, China; b Taizhou Academy of Agricultural Sciences, Taizhou, China; c Joint International Research Laboratory of Agriculture and Agri-Product Safety of the Ministry of Education, Yangzhou University, Yangzhou, China

**Keywords:** Phytosulfokine, PSK-ε, root elongation, lateral root, root nodule, *Medicago truncatula*

## Abstract

Phytosulfokines (PSKs) are a class of tyrosine-sulfated pentapeptides. PSK-α, PSK-γ, and PSK-δ are three reported PSK members involved in regulating plant growth, development, and resistance to biotic and abiotic stresses. Here, we reported a novel type of PSK, PSK-ε with the sequence Y_SO3_VY_SO3_TN, and its precursor proteins (MtPSKε, LjPSKε, and GmPSKε), specifically from legume species. PSK-ε peptide differs from PSK-δ by one amino acid and is close to PSK-δ in the phylogenetic relationship. Expression profile analysis showed that *MtPSKε* was highly expressed in *Medicago truncatula* roots, especially in root tips and emerged lateral roots. Application of the synthetic sulfated PSK-ε peptide and overexpression of *MtPSKε* significantly promoted *M. truncatula* root elongation and increased lateral root number, probably by inducing cell division and expansion in roots. Furthermore, *MtPSKε* expression was induced by rhizobia infection and was detected in root nodules including nodule primordia. Both PSK-ε peptide treatment and *MtPSKε* overexpression significantly increased nodule number in *M. truncatula*. Taken together, these results demonstrate that PSK-ε, a novel type of phytosulfokine, positively regulates root elongation and formation of lateral root and root nodule in *M. truncatula*.

## 1. Introduction

Root system plays an important role in absorption, transport and storage of water and nutrients beyond anchoring and supporting functions. In order to achieve more efficient nutrient acquisition and utilization, root system must flexibly adjust growth and development to adapt to the heterogeneous soil environment.^1^ Internal developmental factors and external environmental signals coordinately shape root system architecture.^2^ Legumes can interact with the *Rhizobium* in the soil to establish a symbiotic relationship, forming a new root-derived organ called root nodule, in which atmospheric dinitrogen is converted to ammonia to support plant growth.^3^ At the molecular level, coordinating plant root development, including root elongation and lateral root formation, and nodulation process are regulated by a complex signaling network. Further investigation of key factors in this network is of great importance to improve the growth and yield of leguminous crops.

Phytosulfokine (PSK) is a kind of tyrosine-disulfated pentapeptide hormone which is widespread across the plant kingdom.^4^ The first identified PSK member is PSK-α with the sequence Y_SO3_IY_SO3_TQ.^5^ A disulfated tetrapeptide with the sequence Y _SO3_IY_SO3_T was also reported and named PSK-β which, however, was found to be a degradation product of PSK-α.^6^ PSK-α peptides are produced from precursor proteins of about 80–120 amino acids encoded by the PSK gene family.^4^ These precursor proteins contain an N-terminal signal peptide sequence and the PSK motif near the C-terminus. PSK-α peptides mature through tyrosine sulfation catalyzed by the tyrosylprotein sulfotransferase (TPST) in the Golgi apparatus^7^ followed by proteolytic cleavage in the apoplast.^8,9^ Mature PSK-α peptides function through a plasma membrane-localized module comprising the receptor kinase PSKR, putative co-receptor BAK1, CNGC17, and H^+^-ATPases.^10,11^ Genetic study and peptide application assay have shown that PSK-α promotes the growth of root, hypocotyl, and leaf by inducing cell expansion or elongation in these organs.^12–14^ It has also been reported that PSK-α induces cell division both *in vivo* and *in vitro*.^5,15^ Moreover, PSK-α also participates in regulating somatic embryogenesis,^16^ drought-induced flower drop,^17^ *in vitro* regeneration of recalcitrant legume,^18^ and immune responses.^19–21^

Our previous work in legumes have identified two analogues of PSK-α peptide, named PSK-γ^22^ and PSK-δ.^23^ PSK-γ is a disulfated pentapeptide with the sequence Y_SO3_VY_SO3_TQ, different from PSK-α only at the second amino acid. Precursor genes encoding PSK-γ peptide are primarily expressed in soybean seeds. Overexpression of PSK-γ genes remarkably promoted seed growth by inducing embryo cell expansion.^22^ PSK-δ is a recently identified disulfated pentapeptide with the sequence Y_SO3_IY_SO3_TN, distinguishing from PSK-α only at the last amino acid. PSK-δ, as a legume species-specific peptide, accumulates primarily in root nodules. Both application of PSK-δ peptide and genetic manipulation showed that PSK-δ promotes *M. truncatula* nodulation by enhancing nodule organogenesis.^23^

In this study, we identified a novel type of PSK, PSK-ε with the sequence Y_SO3_VY_SO3_TN, specifically from legume species. In *M. truncatula*, the PSK-ε-encoding precursor gene *MtPSKε* was primarily expressed in root tips, emerged lateral roots, and root nodules at all developmental stages. Application of the synthetic sulfated PSK-ε peptide and overexpression of *MtPSKε* in *M. truncatula* promoted root elongation and increased numbers of lateral roots and root nodules, probably by inducing cell division and expansion. These findings suggest that PSK-ε positively regulates root elongation and formation of lateral roots and root nodules in legumes and can be exploited to improve crop growth and yield.

## 2. Materials and methods

### 2.1. Plant materials and growth condition

The *Medicago truncatula* Jemalong A17 ecotype and *Arabidopsis thaliana* Columbia-0 ecotype were used for transgenic assays. Seeds of *M. truncatula* were immersed in H_2_SO_4_ for 8 min, rinsed three times with distilled water, surface sterilized with 10% (v/v) NaClO for 3 min and rinsed with distilled water for 6 times. After surface sterilizing, seeds were spread on 1% (w/v) agar medium, and stratified at 4°C for 2 days. Then the seeds were germinated at 28°C in the dark for 12 h. Arabidopsis seeds were surface sterilized for 15 min in 5% (v/v) NaClO solution containing 0.2% (v/v) Tween-20, washed three times with sterilized water, and spread on Murashige and Skoog medium (Duchefa) containing 1% (w/v) sucrose and 0.7% (w/v) agar. Plates were stratified at 4°C for 2 days and then placed in a greenhouse for germination. *M. truncatula* and Arabidopsis seedlings were transplanted to pots containing a 3:1 ratio of vermiculite: perlite and cultured in a greenhouse with a light/dark cycle of 16 h/8 h at 23°C and 50% relative humidity.

### 2.2. qRT-PCR gene expression analysis

The plant tissues were homogenated in liquid nitrogen, and the total RNA was isolated using RNAprep pure plant kit (Tiangen, Beijing, China). A NanoDrop spectrophotometer (Thermo Fisher, Waltham, MA, USA) was used to quantify the RNA concentration and the first-strand cDNA was synthesized using the HiScript II Q-RT Supermix kit (Vazyme, Nanjing, China). For gene expression analysis, qRT–PCR experiments were carried out on a CFX96 real-time PCR detection system (Bio–Rad, Hercules, CA, USA) with Hieff qPCR SYBR Green Master Mix (Yeasen, Shanghai, China). PCR conditions and data analysis methods were described in our previous study.^24^ The housekeeping genes used in qRT–PCR experiments were *MtActinB* or *AtActin2*, and the specific primers are listed in Table S1.

### 2.3. Gene cloning and vector construction

For promoter-GUS assay, the promoter region (approximately 2000 bp upstream of start codon) of *MtPSKε* was cloned from *M. truncatula* genomic DNA by PCR and ligated into the binary vector pBI121 to drive the expression of the *GUS* (β-glucuronidase) gene. For Overexpression constructs, the *MtPSKε* CDS was PCR amplified from *M. truncatula* cDNA, and was ligated into the intermediate vector pA7 downstream of the Cauliflower mosaic virus (CaMV) 35S promoter using the restriction sites XhoI and SacI. The resulting *35S:MtPSKε:OCSter* cassette was subsequently ligated into a modified binary vector pCambia1300 using the restriction sites HindIII and EcoRI. The modified pCambia1300 vector contains a *35S:GFP* module in the T-DNA region, facilitating GFP fluorescence screening of transgenic hairy roots. The primers for the constructs are listed in Table S1.

### 2.4. Exogenous peptide treatment

PSK-ε (Y_SO3_VY_SO3_TN) peptide and randomly arranged pentapeptide (TYNYV) were synthesized by Chinese Peptide Company (Hangzhou, China). Each peptide was stored at 1 mM in sterilized double-distilled water as a stock solution. To investigate the effect of PSK-ε on root growth, *M. truncatula* seedlings were grown on FM plates containing 1 μM corresponding synthetic peptides. Seedlings were photographed and the lateral roots were counted. For the nodulation assay, germinated seeds of *M. truncatula* were grown on FM plates supplemented with corresponding peptides at a concentration of 1 μM, and *S. meliloti* 2011 was suspended in FM liquid solution (OD_600_ = 0.02) and flood-inoculated onto roots. After 20 days of growth on plates in the vertical position, the nodules were photographed and counted.

### 2.5. Hairy root transformation

For hairy root transformation, the *35S:MtPSKε* plasmid were introduced into the *A. rhizogenes* ARqua1 strain. The recombinant Arqua1 was used to transform *M. truncatula* roots according to a well established method.^25^ The positive transgenic roots were selected based on GFP fluorescence examined under a SMZ18 fluorescence stereomicroscope (Nikon, Tokyo, Japan). The transgenic plants were transplanted to pots and inoculated with *S. meliloti* 2011. After 3 weeks of growth, the nodulation phenotype was analyzed.

## 3. Results

### 3.1. Sequence and phylogeny of legume-specific PSK-ε precursor proteins

**Figure 1.**
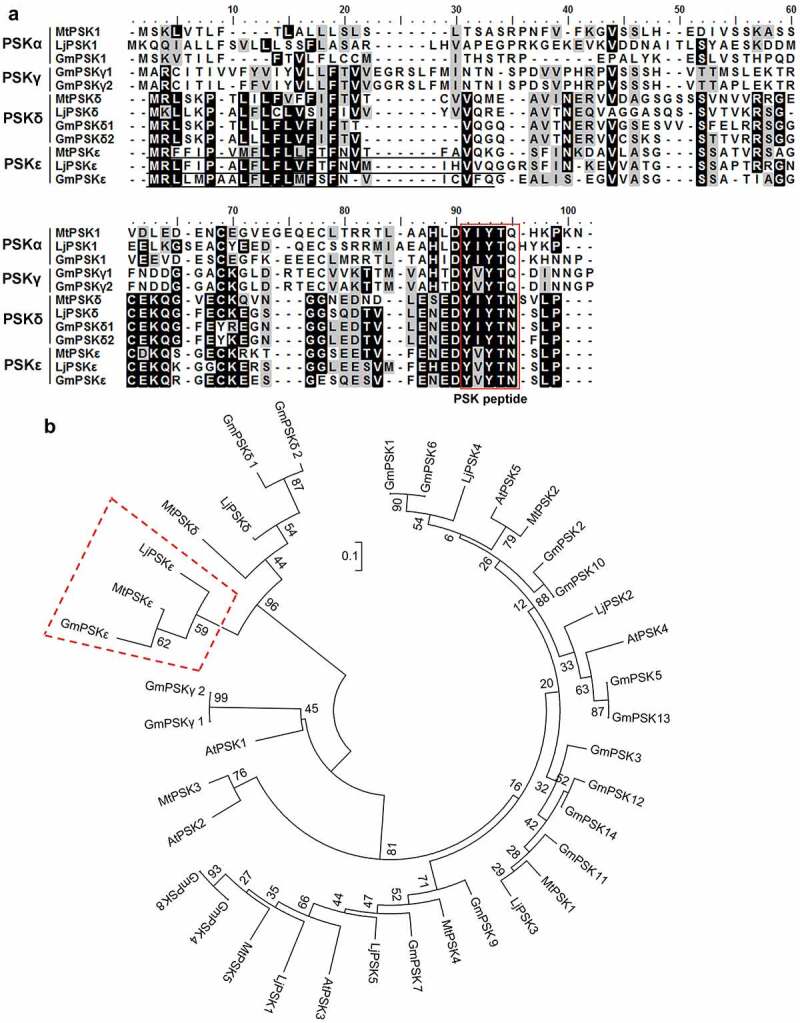
Legume-specific *PSKε* genes are predicted to encode a novel type of phytosulfokine precursor protein. (a) Sequence alignment of representative PSKα, PSKγ, PSKδ, and predicted PSKε precursor proteins in *Medicago truncatula, Lotus japonicus*, and *Glycine max*. Conserved and similar amino acid residues are shaded in black and gray, respectively. The PSK pentapeptide motifs near the C-terminus of PSK precursors are boxed in red. The predicted signal peptide sequences at the N-terminus are underlined. (b) Phylogenetic tree of PSKα, PSKγ, PSKδ, and PSKε members in Arabidopsis and three legume species. Legume-specific PSKε precursor proteins cluster in one phylogenetic clade, as indicated by a red dashed-line box. The tree was constructed using MEGA6 software with the neighbor-joining method, and bootstrap values from 1000 replications are included.

Through searching genome databases of legume species (phytozome-next.jgi.doe.gov) using reported PSK precursor sequences as queries, we identified a class of legume-specific genes encoding putative PSK pentapeptides. These predicted pentapeptides with the sequence YVYTN differ from PSK-γ (YVYTQ) by the last amino acid (substitution of glutamine by asparagine), and differ from PSK-δ (YIYTN) by the second amino acid (substitution of isoleucine by valine) (Figure 1a), therefore, the new peptide is named PSK-ε in accordance with the naming convention. Similar to PSK-δ, PSK-ε exists only in legume species. In the genomes of *M. truncatula* (A17), *L. japonicus* (MG-20) and *G. max* (Williams 82), one gene was found to encode the PSK-ε precursor protein, named *MtPSKε* (Medtr8g091700), *LjPSKε* (Lj4g0007102) and *GmPSKε* (Glyma.08G140800), respectively (Figure 1a). Like the reported PSK members, all these PSK-ε precursor genes contain two exons (Fig. S1), and the deduced proteins consist of approximately 80 amino acids. Sequence alignment showed that these PSK-ε precursors were highly conserved (78–85% sequence similarity), and were more similar with PSK-δ than with PSK-α and PSK-γ (Figure 1a). As predicted by SignalP-5.0 (www.cbs.dtu.dk/services/SignalP/), all PSK-ε precursors harbor N-terminal signal peptide sequences for targeting the secretory pathway, the PSK-ε pentapeptide motifs and the conserved aspartic acid at the −1 position of pentapeptide were identified at the C-terminus (Figure 1a).

Phylogenetic analysis revealed that the legume-specific PSK-ε precursor proteins MtPSKε, LjPSKε, and GmPSKε have a closer relationship with PSK-δ proteins, which are also legume-specific. PSK-ε and PSK-δ members are clustered into one clade (Figure 1b), forming a PSK sub-family. These results indicate that, like PSK-δ, PSK-ε has a specific function in legume growth and development.

### 3.2. Expression pattern of PSK-ε precursor genes in *M.* *truncatula*

**Figure 2.**
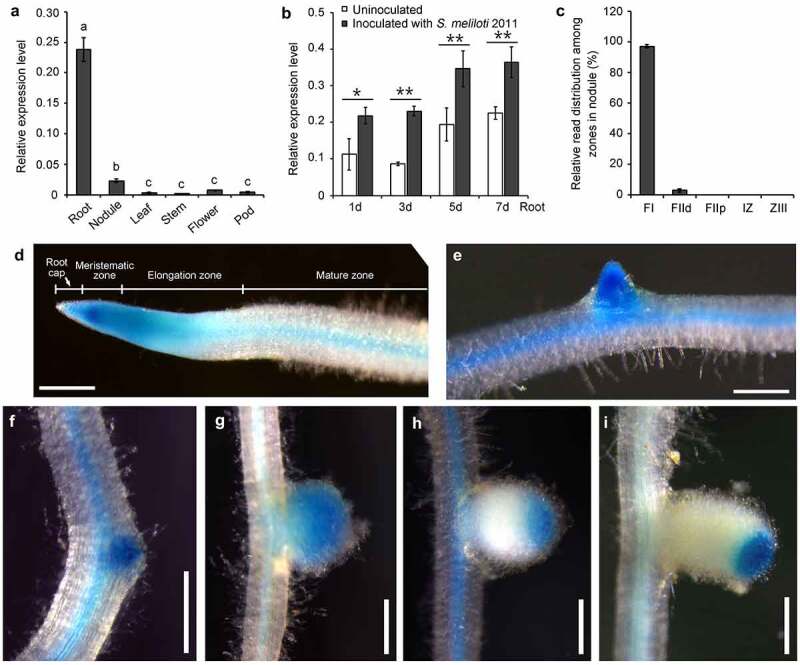
Expression pattren of *MtPSKε* in *M. truncatula*. (a) Expression pattern of *MtPSKε* determined by qRT–PCR. cDNA was prepared from wild-type *M. truncatula* (A17 ecotype) organs, including roots, root nodules (14 d post-inoculation, 14 dpi), leaves, stems, flowers, and pods. qRT–PCR was employed to detect *MtPSKε* transcript levels in the various tissues. Expression of *MtActinB* was used as an internal control. Statistically significant differences indicated by different letters were determined with one-way ANOVA (P < .05) (b) Expression of *MtPSKε* in *M. truncatula* roots inoculated with *S. meliloti* 2011 at different times (1–7 dpi) with expression in uninoculated roots as controls. Statistical significance was evaluated by Student’ s *t* test; * P < .05, ** P < .01. (c) Expression level of *MtPSKε* in different regions of developing nodules at 15 dpi. FI, meristematic zone; FIId, distal infection zone; FIIp, proximal infection zone; IZ, interzone; ZIII, nitrogen-fixation zone. The data was collected from published RNA-Seq data (https://iant.toulouse.inra.fr/symbimics/). Values represent the means ± SE of three biological replicates. (d-i) Histochemical staining of *pMtPSKε:GUS* transgenic hairy roots of *M. truncatula* (A17). GUS activity detected in transgenic primary roots (d) and emerged lateral roots (e). GUS staining of root nodule primordia at 4 dpi (f), young nodules (7 dpi, G), elongating nodules (12 dpi, H), and mature nodules (20 dpi, I). Scale bars = 500 μm.

To investigate the expression pattern of the PSK-ε precursor gene *MtPSKε*, cDNA from various organs of *M. truncatula* were prepared for quantitative RT-PCR assay. The results showed that *MtPSKε* was highly expressed in roots and weakly in nodules, while in other organs (leaf, stem, flower, and pod), was expressed at extremely low levels (Figure 2a). To investigate whether *MtPSKε* is regulated by rhizobia infection, the transcription level of *MtPSKε* was examined in *M. truncatula* roots at 1, 3, 5, and 7 d post-inoculation (dpi) with *S. meliloti* 2011. As shown in Figure 2b, *MtPSKε* expression was upregulated upon rhizobia inoculation at an early stage (1 dpi) and each stage followed, indicating that *MtPSKε* expression in root is induced by rhizobia. Additionally, published data of nodule laser capture micro-dissection coupled to RNA-seq showed that *MtPSKε* is predominantly expressed in the apical meristem and partial infection zone of developing *M. truncatula* nodules (Figure 2c).

To further determine the spatiotemporal expression of *MtPSKɛ*, we cloned the promoter of *MtPSKɛ* and fused it with the *GUS* coding gene. The resulting *pMtPSKɛ:GUS* construct was transformed into *M. truncatula* hairy roots mediated by *Agrobacterium rhizogenes*. Histochemical staining showed that *pMtPSKɛ:GUS* was expressed in the tip of primary roots (Figure 2d), emerged lateral roots (Figure 2e), and root vascular tissues (Figure 2d, e). In root tip, the strongest GUS activity was detected in the meristematic zone and lower activity in elongation zone and root cap (Figure 2d). Moreover, *pMtPSKɛ:GUS* was expressed throughout the root nodule developmental process. Strong GUS signal was detected as early as in the nodule primordia (Figure 2f), and was persistently detected in the whole young nodules (Figure 2g), where active cell division and expansion occurred. In elongating (Figure 2h) and mature (Figure 2i) nodules, GUS signal was restricted to the apical regions, corresponding mainly to meristematic and partial infection zones, consistent with the findings in Figure 2c. These results demonstrate that *MtPSKɛ* gene is mainly expressed in root and nodule regions where cell division and expansion are active, suggesting a key role of PSK-ɛ peptide in regulating root and nodule growth and development.

### 3.3. PSK-ε peptide promotes root system development

**Figure 3.**
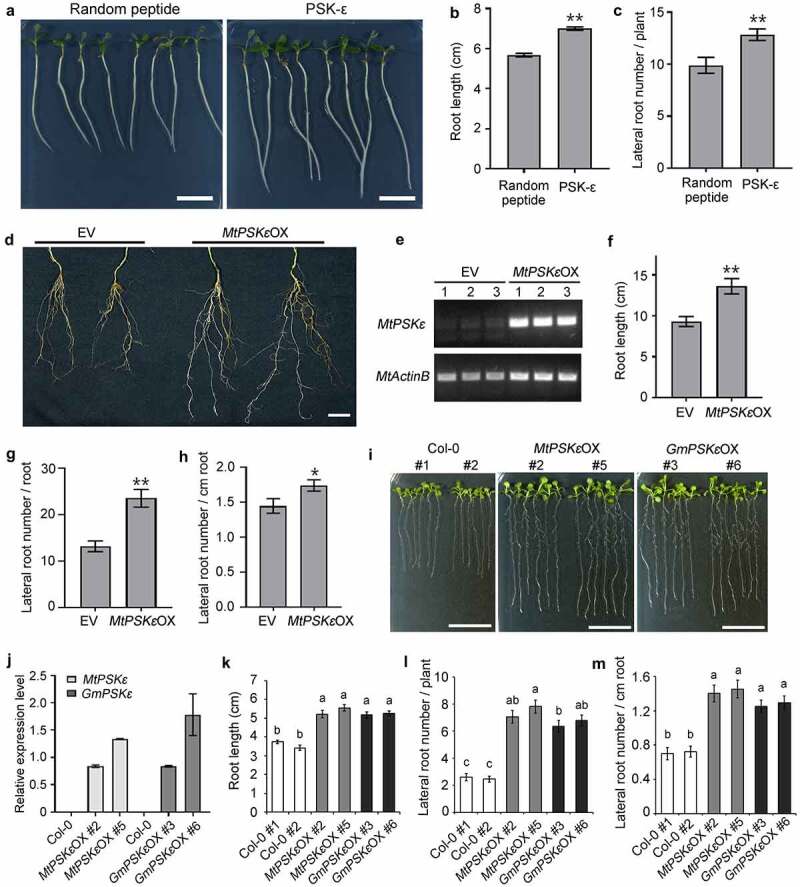
PSK-ε peptide promotes root development. (a-c) Exogenous application of synthetic PSK-ε peptide in *M. truncatula*. (a) Seven-day-old *M. truncatula* (A17) seedlings grown on vertical FM plates containing 1 μM PSK-ε peptide or 1 μM randomly arranged pentapeptide. Scale bars = 2 cm. (b) Primary root length of *M. truncatula* seedlings treated with PSK-ε or random peptide for 7 days (n = 25). (c) Lateral root number of PSK-ε and random peptide-treated *M. truncatula* plants at 20 days of age (n = 20). (d-h) Overexpression of *MtPSKε* in *M. truncatula* (A17) transgenic hairy roots. (d) Comparison of *MtPSKε* overexpressing (*MtPSKε*OX) transgenic roots and the empty vector (EV) control roots at 20 d post-inoculation (dpi). Scale bar = 2 cm. (e) Semi-quantitative RT–PCR analysis of *MtPSKε* transcript abundance in underground tissues (including roots and nodules) of the control and *MtPSKε*OX plants at 20 dpi. Expression of *MtActinB* was used as an internal control. (f) Root length of the *MtPSKε*OX and control plants at 20 dpi. (g) Number of lateral roots formed on 20 dpi *MtPSKε*OX and control transgenic roots. (h) Lateral root number per unit length (cm) of the transgenic roots. In (f-h), n = 24 for *MtPSKε*OX, n = 25 for empty vector control. (i-m) Heterologous overexpression of *MtPSKε* or *GmPSK*ε promotes root growth in Arabidopsis. (i) Eight-day-old wild-type and transgenic Arabidopsis seedlings grown on vertical MS-agar plates. Col-0 seeds from two stocks were used as wild-type controls. Scale bars = 2 cm. (j) qRT-PCR analyses of *MtPSKε* and *GmPSKε* transcript levels in *MtPSKε* and *GmPSKε*-overexpressing Arabidopsis lines. Values are the means ± SE of three independent biological replicates normalized against the reference gene *AtActin2*. (k) Primary root length of the wild-type and transgenic Arabidopsis lines showed in (I). (l) Number of lateral roots formed on the wild-type and transgenic Arabidopsis lines showed in (I). (m) Lateral root number per unit length (cm) of the wild-type and transgenic Arabidopsis roots. Values of root length and lateral root number are means ± SE. n > 25. In (B, C, F, G, H), statistical significance was evaluated by Student’ s t test; * P < .05, ** P < .01. In (k-m), statistically significant differences indicated by different letters were determined with one-way ANOVA, P < .05.

To investigate the biological function of PSK-ε peptide in plant growth and development, we supplied 1 μM chemically synthesized PSK-ε pentapeptide exogenously to wild-type *M. truncatula* seedling grown on vertical plates. Seedlings treated with randomly arranged pentapeptide at the same concentration were used as a control. After 7 days of growth, we found that the length of primary root treated with PSK-ε is 23% longer than that of the random peptide treatment (Figure 3a, b). After 20 days of growth, the PSKε-treated *M. truncatula* plants generated more lateral roots compared to the random peptide control (Figure 3c).

To further determine the effect of PSK-ε on growth and development of the root system, we constructed *MtPSKε* overexpression vector driven by the enhanced cauliflower mosaic virus (CaMV) 35S promoter and transformed into *M. truncatula* hairy roots mediated by *Agrobacterium rhizogenes*. Semi-quantitative RT-PCR showed that *MtPSKε* was successfully overexpressed in transgenic hairy roots compared with the empty vector transformation roots (Figure 3e). Phenotypic observation revealed that the *MtPSKε*-overexpressing transgenic plants developed better root system than did the empty vector control (Figure 3d). Specifically, the length of primary roots (figure 3f) and the number of lateral roots (Figure 3g) increased by 46% and 78%, respectively; and the lateral root density was also increased (Figure 3h). These results coincide with the findings of exogenous peptide treatments.

Additionally, we constructed constitutive expression vectors of *MtPSKε* and *GmPSKε* under the control of 35S promoter, and transformed into Arabidopsis mediated by *Agrobacterium tumefaciens*. For each gene, more than 16 homozygous transgenic lines (T3 generation) were obtained. Among these lines, *MtPSKε*-overexpressing lines 2 and 5, and *GmPSKε*-overexpressing lines 3 and 6 were highly expressed (Figure 3j). For phenotypic observation, overexpression lines and wild-type Arabidopsis were grown on vertical plates for 8 days. The results showed that both *MtPSKε* and *GmPSKε* overexpression significantly increased root length (Figure 3i, k), number of lateral roots per plant (Figure 3i, l), and density of lateral roots (Figure 3m). Microexamination revealed that expression of *MtPSKε* or *GmPSKε* in Arabidopsis increased root meristem size and cell length, leading to longer roots (Fig. S2). Therefore, PSK-ε positively regulates the root development by promoting root growth and lateral root formation in plants.

### 3.4. PSK-ε promotes nodulation in *M.* *truncatula*

**Figure 4.**
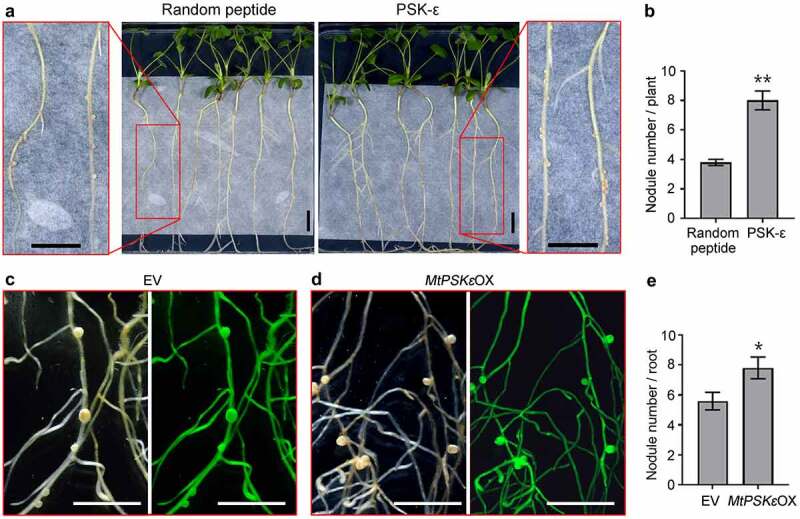
PSK-ε peptide promotes root nodule formation in *M. truncatula*. (a) *M. truncatula* (A17) seedlings inoculated with *S. meliloti* 2011 for 20 days were grown on FM agar plates supplemented with 1 μM synthetic peptides (PSK-ε or random peptide). (b) Statistic analysis of the nodule number on PSK-ε and random peptide-treated *M. truncatula* seedlings (n = 20). (c, d) Nodulation phenotype of empty vector (c) and *MtPSKε*-overexpressing (d) transgenic *M. truncatula* roots at 20 dpi of *S. meliloti* 2011. Left panels, bright-filed images; right panels, fluorescence images. (e) Numbers of nodules formed on 21 dpi transgenic *M. truncatula* roots (n = 27). Scale bars = 1 cm in (A), (C), and (D). In (B) and (E), values are means ± SE, statistical significance was evaluated by Student’ s t test; * P < .05, ** P < .01. Experiments were repeated three times with similar results.

Based on the findings that *MtPSKε* expression is induced by rhizobia and is throughout the root nodule developmental process, it is reasonable to presume that PSK-ε is involved in the regulation of nodule formation in legumes. To test this assumption, we supplied 1 μM synthesized PSK-ε peptide or randomly arranged pentapeptide exogenously to *M. truncatula* seedling grown on vertical nitrogen-free plates, and then inoculated them with *S. meliloti*. At 20 dpi, the plants treated with PSK-ε peptide formed approximately one-fold more nodules than did the control plants (Figure 4a, b). To validate the function of PSK-ε in nodulation, *35S:MtPSKε* construct was transformed into *M. truncatula* hairy roots. At 20 dpi of *S. meliloti*, phenotypical observation revealed that although the shape and size of nodules were not obviously altered, the number of nodules on *MtPSKε*-overexpressing transgenic roots was increased by 39% relative to control roots (Figure 4c-e). These results demonstrate that PSK-ε peptide positively regulates root nodule formation.

## 4. Discussion

PSK peptides are widespread across the plant kingdom. The first member PSK-α and its precursor genes have been extensively identified and studied in multiple plant species, revealing their important roles in plant growth, development, and innate immunity.^4^ Recently, we have identified two new PSK members, PSK-γ and PSK-δ, specifically in legume species. Both PSK-γ and PSK-δ pentapeptides have one amino acid different from the PSK-α peptide and were viewed as analogues of the latter.^22,23^ PSK-γ was primarily expressed in soybean seeds, and overexpression of its precursor gene remarkably increased seed size through inducing cell expansion;^22^ while PSK-δ peptide, accumulated mainly in legume nodules, promotes symbiotic nodulation by enhancing nodule organogenesis.^23^ In this study, we identified a novel type of PSK, PSK-ε, specifically in legume species. Similar to the other PSK members, PSK-ε precursors have a typical protein length and contain N-terminal signal peptide and PSK-ε pentapeptide motifs near the C-terminus. Additionally, PSK-ε differs from PSK-δ peptide only at the second amino acid (the isoleucine in PSK-δ substituted by a valine in PSK-ε). Given that isoleucine and valine are similar in chemical characteristics, it is reasonable to presume that the two types of PSKs share similar biochemical activities. On the other hand, PSK-ε has a closer phylogenetic relationship with PSK-δ than with the other two PSK members. These findings strongly indicate that PSK-ε is a functional analogue of PSK-δ.

Promoter:GUS assay showed that *MtPSKε* gene was primarily expressed in root tips, emerged lateral roots, and root nodules at all developmental stages, which is similar with the expression profile of *MtPSKδ* revealed by GUS staining.^23^ Although the two PSK members exhibit similar expression positions in under-ground tissues, the expression abundance, however, is different. As revealed by qRT-PCR experiments, *MtPSKε* is highly transcribed in root system and much lower in nodules. Conversely, *MtPSKδ* is primarily expressed in nodules and only a weak transcript level is detected in roots.^23^ This differential expression pattern indicates that PSK-ε primarily regulates root growth and development while PSK-δ is mainly involved in nodule formation. Actually, both application of PSK-ε peptide and overexpression of *MtPSKε* significantly increased root elongation and lateral root number in *M. truncatula*, and even heterologous expression of *MtPSKε* or *GmPSKε* in Arabidopsis remarkably promoted root growth and lateral root formation. In contrast, PSK-δ exhibited only a weak promotion effect on root growth and development.^23^ Furthermore, GUS staining demonstrated that, in *M. truncatula* root system, *MtPSKε* was heavily expressed in meristematic and elongation zones of root tip and in emerged lateral roots where rapid cell division and expansion occurred, indicating that PSK-ε peptide induces cell division and expansion to promote root elongation and lateral root formation. Actually, heterologous expression of *MtPSKε* or *GmPSKε* in Arabidopsis induced both cell division and elongation to promote root growth. This mechanism is reminiscent of the previous findings that the other three PSK members function through inducing cell proliferation or cell growth.^12–15,22,23^ These results demonstrate that as a new PSK member, PSK-ε has similar biochemical activity with the reported PSKs.

In this study, we also found that *MtPSKε* expression was induced by rhizobia infection and was detected throughout the whole root nodule developmental process. Accordingly, both Application of PSK-ε peptide and overexpression of *MtPSKε* significantly increased nodule number in *M. truncatula*. Given that *MtPSKε* was expressed as early as in the nodule primordia, it is conceivable that PSK-ε peptide might positively regulate primordium initiation to enhance nodule formation. Our previous work reported that PSK-δ promotes *M. truncatula* nodulation by inducing nodule organogenesis.^23^ These results suggest that PSK-ε and PSK-δ peptides regulate legume nodulation in a similar mechanism.

## Supplementary Material

Supplemental Material
