# Supplementary material for: A novel type of phytosulfokine, PSK-ε, positively regulates root elongation and formation of lateral roots and root nodules in Medicago truncatula
Source: Plant Signal Behav. 2022 Nov 10;17(1):2134672. doi: 10.1080/15592324.2022.2134672 (PMC9662189; doi:10.1080/15592324.2022.2134672)
Supplement: Supplemental Material [file KPSB_A_2134672_SM6872.zip › Table S1.docx]

| **Gene** | **Forward primer (5’-3’)** | **Reverse primer (5’-3’)** | **Application** |
| --- | --- | --- | --- |
| *MtPSKε* | TCTCTTATTTACCTTCAATGTG | CCCAGCACTTCTAACAGTGG | qRT-PCR |
| *GmPSKε* | AGACTATTGATGCCTGCTGC | GCCACCTGCAATAGTAGCAG | qRT-PCR |
| *MtActinB* | AACTGGAATGGTCAAGGCTGG | TATAGGATACTTCAACGTGAGG | qRT-PCR |
| *AtActin2* | AACCGGTATTGTGCTGGATTC | AGGTTTCCATCTCCTGCTCG | qRT-PCR |
| *MtPSKε* | CCCAAGCTTACAAGGACTAACTAGGCGG | CGGGATCCTGTATTAGACCTAACTAGC | Promoter-GUS |
| *MtPSKε* | CAGCGGATCCATGAGATTTTTTATACCTGT | CTACGAGCTCCTAAGGTAAAGAGTTGGTAT | Overexpression |
| *MtPSKε* | GCGCTCGAGATGAGATTTTTTATACCTGT | GCGGAGCTCCTAAGGTAAAGAGTTGGTAT | Heterologous overexpression |
| *GmPSKε* | GCACTCGAGATGAGACTATTGATGCCTGC | GCGGAGCTCCTAAGGCAAGGAGTTGGTAT | Heterologous overexpression |
