# Supplementary figures and images for: A novel type of phytosulfokine, PSK-ε, positively regulates root elongation and formation of lateral roots and root nodules in Medicago truncatula
Source: Plant Signal Behav. 2022 Nov 10;17(1):2134672. doi: 10.1080/15592324.2022.2134672 (PMC9662189; doi:10.1080/15592324.2022.2134672)

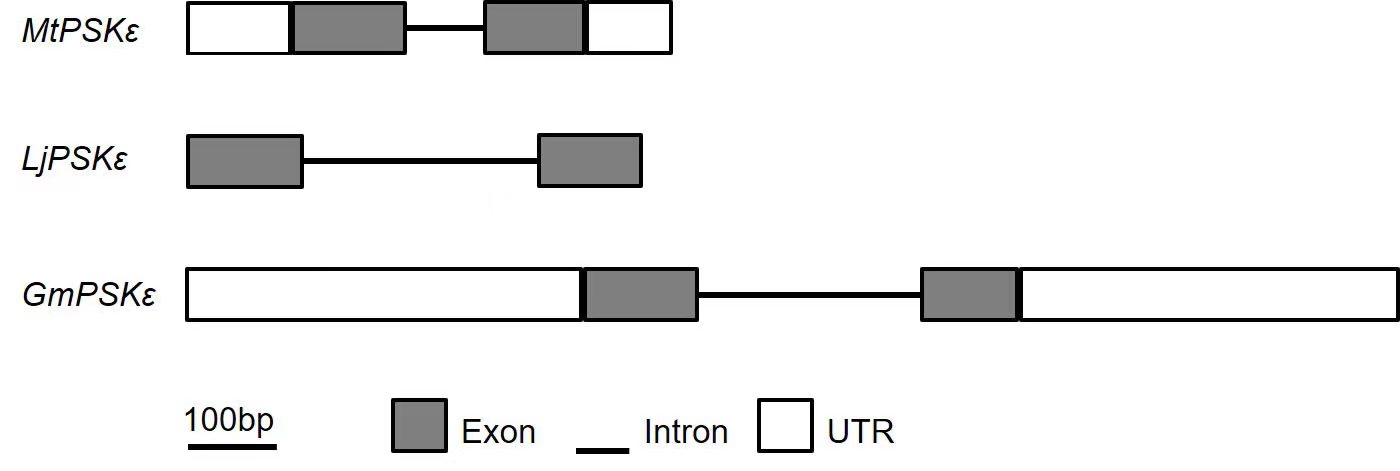

Supplement: Supplemental Material [file KPSB_A_2134672_SM6872.zip › Figure S1.jpg]

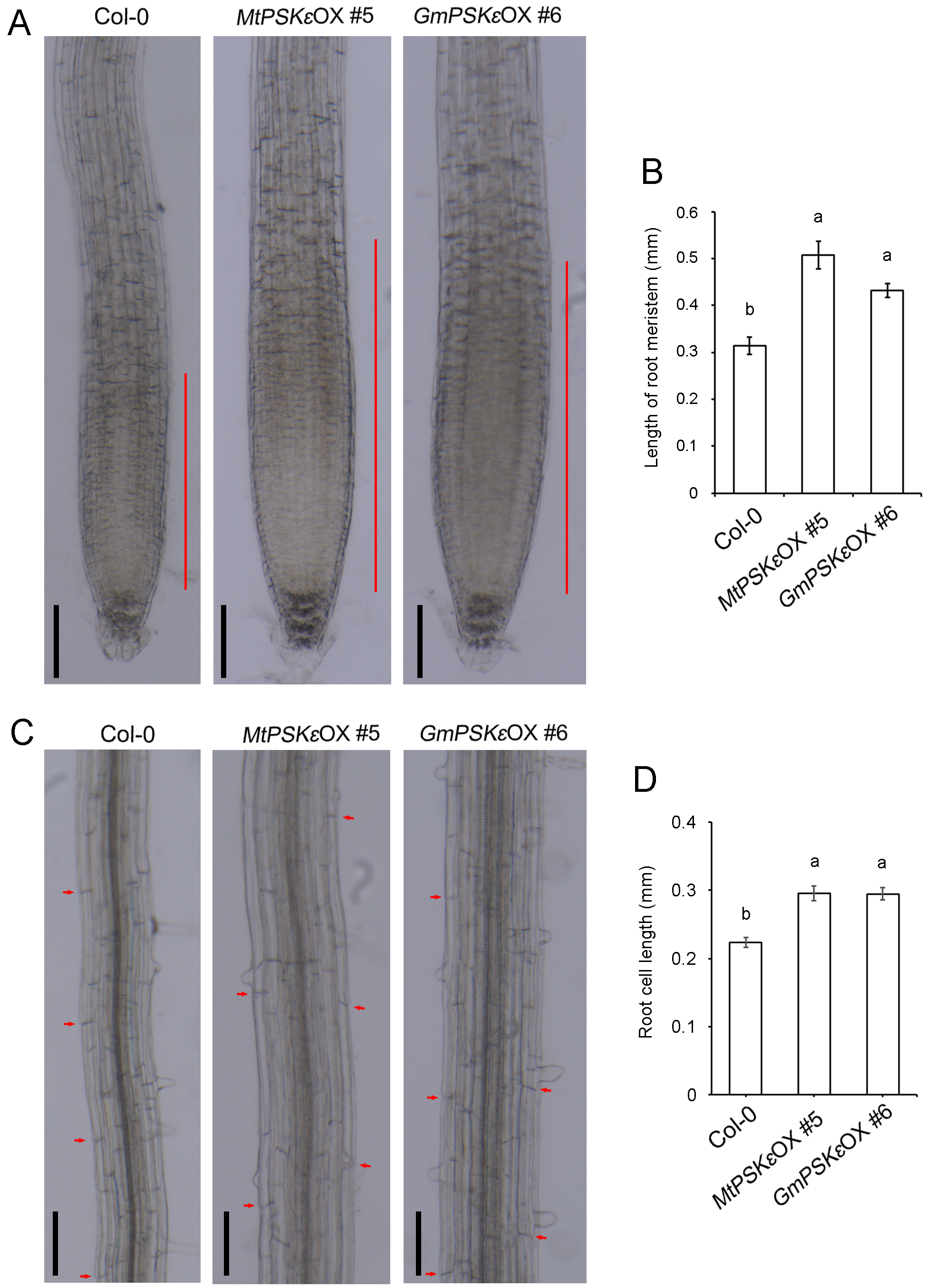

Supplement: Supplemental Material [file KPSB_A_2134672_SM6872.zip › Figure S2.jpg]
